# Evaluation of the Interest in and Tolerance of a Topical Emollient in the Management of Canine Nasal Hyperkeratosis: An Open-Label, Prospective, Uncontrolled Pilot Study †

**DOI:** 10.3390/vetsci12090792

**Published:** 2025-08-22

**Authors:** Sébastien Viaud, Sarah Pariente, Bruno Jahier, Christelle Navarro, Cécile Destaing, Carole Gard

**Affiliations:** 1 CHV AniCura Aquivet, 4 Rue Léon Morane, 33700 Mérignac, France; 2 MP Labo, 45 Boulevard Marcel Pagnol, 06130 Grasse, France

**Keywords:** nasal, hyperkeratosis, dog, moisturizer, emollient balm

## Abstract

(1) Background: Idiopathic or familial nasal hyperkeratosis (NHK) may be considered a cosmetic issue in its uncomplicated form. Nevertheless, the prevention of secondary lesions could be advised by proper management. (2) Methods: In this multicentre, prospective, open-label pilot study, dogs with familial or idiopathic NHK received a topical emollient balm twice daily for 60 days. Evaluation of the global dermatological score (GDS; 0–12) on D0, D30, and D60 by owners and investigators was the main outcome. The score’s percentage decrease versus D0; the evolution of “dryness”, “lichenification”, “crusts”, and “affected area” (0–3); the correlation between observers and the evaluation of clinical improvement; and animal discomfort rated using a VAS (0–10) during follow-up were analysed. (3) Results: Twenty dogs completed the study. The mean GDS decreased significantly from Day 0 to Day 30 and Day 60 (*p* < 0.0001), with percentage reductions of 44.9% and 54.5% (investigators) and 54.3% and 62.3% (owners) on Day 30 and Day 60, respectively. Correlations between investigators and owners were significant for dryness, crusts (at Day 60), and the affected area, but not for lichenification. Animal discomfort scores decreased by more than 50% from Day 0. Owners reported high satisfaction with the product (mean score 8.1/10) and no adverse events occurred. (4) Conclusions: This open-label pilot study suggests that the tested balm is well tolerated and may improve clinical signs of NHK in dogs. Given the lack of a control group, small sample size, and absence of blinding, these results should be considered preliminary. Further randomized controlled trials and formal validation of the scoring system are warranted.

## Simple Summary

Nasal hyperkeratosis (NHK) is a common dermatological condition in dogs, particularly in brachycephalic breeds, that can lead to cosmetic concerns, discomfort, and secondary infections. Available management options are limited and poorly documented. This open-label, prospective, uncontrolled clinical trial evaluated the efficacy and safety of a new topical emollient balm (Sensiderm^®^ Balm, MP Labo, France) in 20 dogs with familial or idiopathic NHK. A novel scoring system was used to assess lesion severity. After 60 days of twice-daily application, both investigators and owners observed marked improvements in nasal lesions and discomfort without adverse effects. Owners reported high satisfaction with the ease of use and effectiveness of the product. These preliminary findings support the balm’s potential as part of NHK management and justify further controlled studies.

## 1. Introduction

Hyperkeratosis is described as an increased thickening of the stratum corneum and remains mainly associated with epidermal hyperplasia [1]. When affecting only the nasal planum of otherwise healthy dogs, genetic or idiopathic causes are suspected after excluding other conditions such as bacterial or fungal infections (cutaneo-mucous pyoderma dermatophytosis), autoimmune diseases (discoid lupus erythematosus, pemphigus, and Vogt–Koyanagi–Harada syndrome), or neoplasia (nasal lymphoma) [2].

A hereditary nasal dermatitis was described in Golden Retrievers and Greyhounds, where hyperkeratotic lesions were noticed between 6 and 12 months of age. The lesions varied in severity from mild to severe and specifically affected the nasal planum. They consisted of dry and rough areas of brown to grey adherent keratin with variable depigmentation [3,4]. Histopathology showed a modified parakeratotic cornification process and the disappearance of the granular aspect of the corneocyte cytoplasm and lamellar bodies [4]. An important intercellular accumulation of proteinaceous fluid [4], also referred to as a “serum lake” [5], was described as well. In both breeds, the disorder may be inherited by monogenic autosomal recessive transmission. A mutation located in the SUV39H2 gene encoding a histone 3 lysine 9 methyltransferase may be responsible for this hereditary nasal parakeratosis (HNPK). This chromatin-modifying enzyme may indeed interfere with keratinocyte differentiation [6,7,8].

Such a syndrome was also observed in Belgian Griffons [9], in this case, lamellar orthokeratotic hyperkeratosis. Fissures, circumscribed erosions, and mild to moderate depigmentation were also present.

Abnormal anatomy and keratin build-up may predispose brachycephalic breeds to nasal hyperkeratosis (NHK) but little has been published on the subject [9].

Although mild changes of the nasal planum may initially seem cosmetic, they can progress to painful fissures or erosions that predispose dogs to secondary infections. Management of NHK, whether idiopathic or familial, is currently poorly documented and essentially aims at topical moisturization [7]. Several authors reported that topical applications of propylene glycol in water, white petrolatum/petroleum jelly, or topical vitamin E have been reported to improve lesions temporarily, but continuous application is required to maintain the effects [4,5]. A recently published study demonstrated the benefits in addressing the clinical signs of NHK in dogs of a balm composed of the essential oil of *Melaleuca cajuputi*, soybean oil, allantoin, and cetyl palmitate, containing no preservatives or petroleum jelly [10]. The objectives of this study were (i) to evaluate, in an open-label pilot setting, the clinical interest in and tolerance of a novel emollient balm containing urea, glycerin, shea butter, panthenol, and Centella asiatica extract (Sensiderm^®^ Balm, MP Labo, France) in dogs with idiopathic or hereditary NHK, and (ii) to test a newly developed scoring system applicable both by veterinarians and owners.

## 2. Materials and Methods

In this multicentre, prospective, open-label pilot trial, all dogs were recruited at the investigators’ practices in France. The study was conducted according to Good Clinical Practices (—VICH Topic GL9 (GCP)—CVMP/VICH/595/98—4 July 2000); therefore, no ethical approval was required. Owners were asked to sign an informed consent form before their dogs’ enrolment.

### 2.1. Study Population

**Figure 1:**
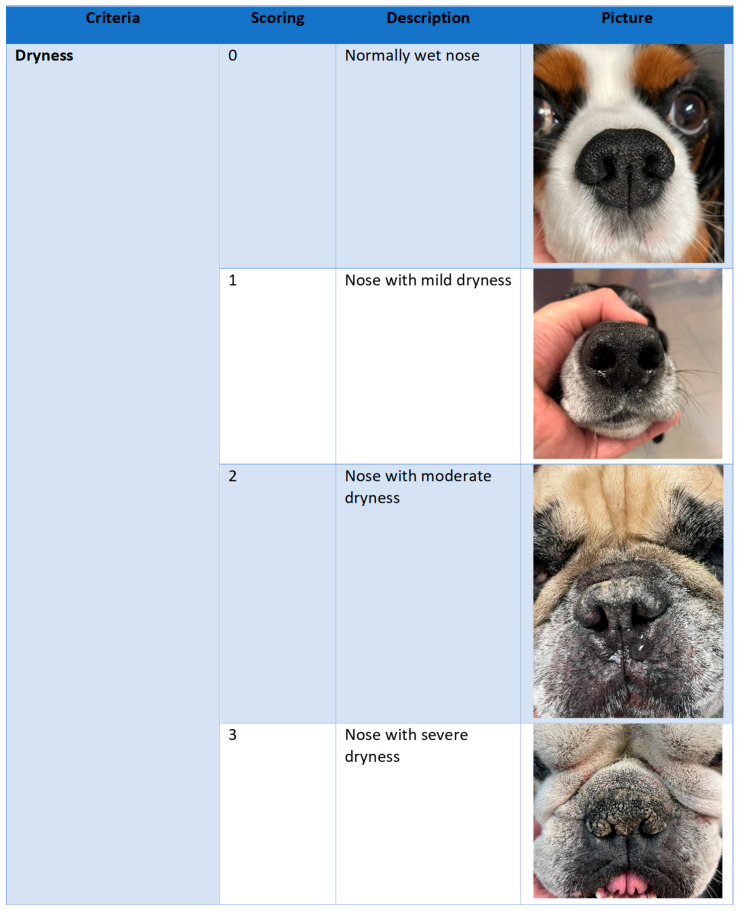

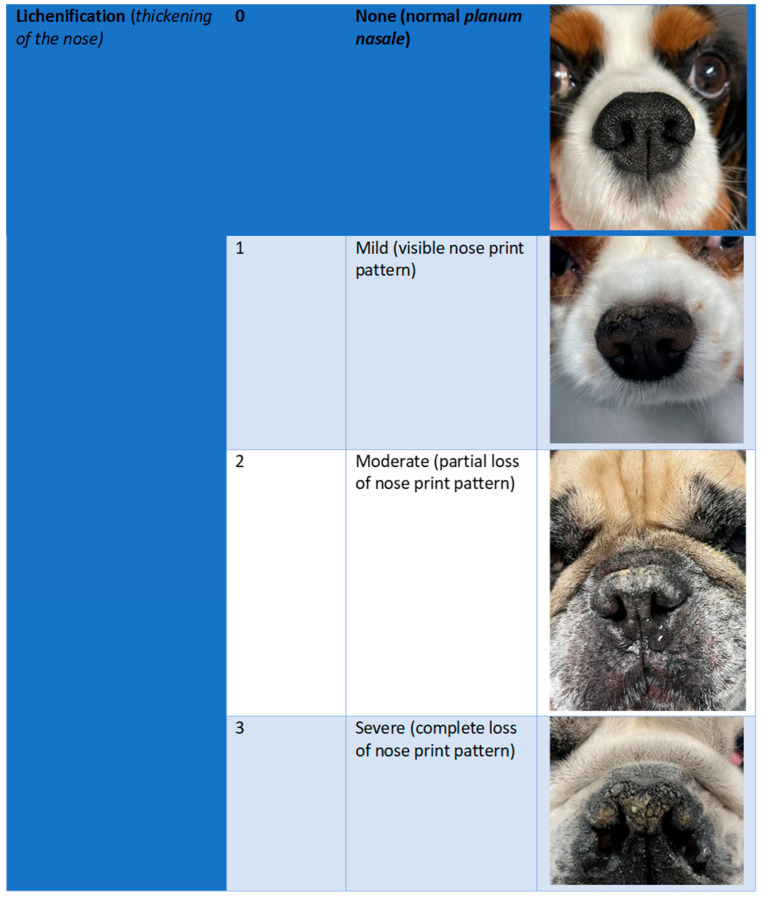

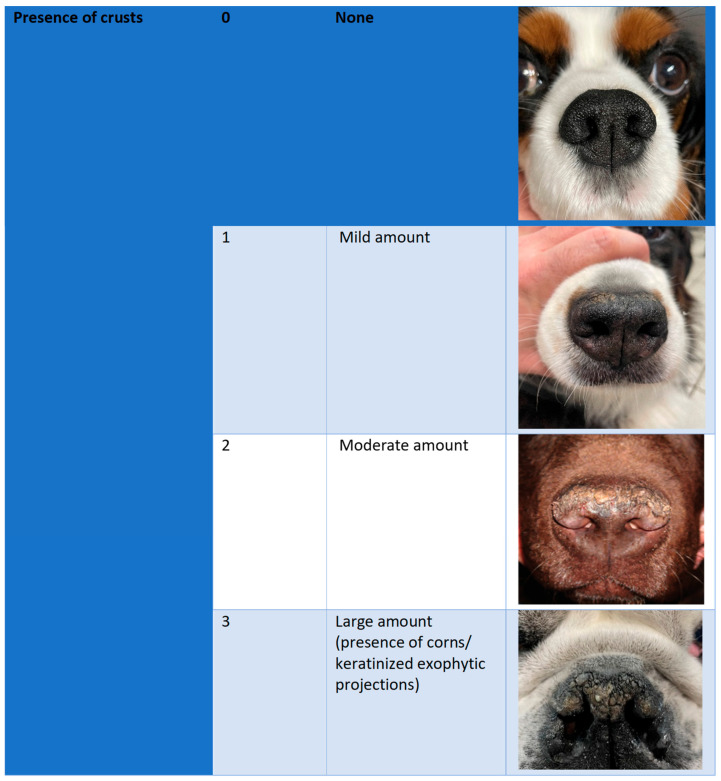

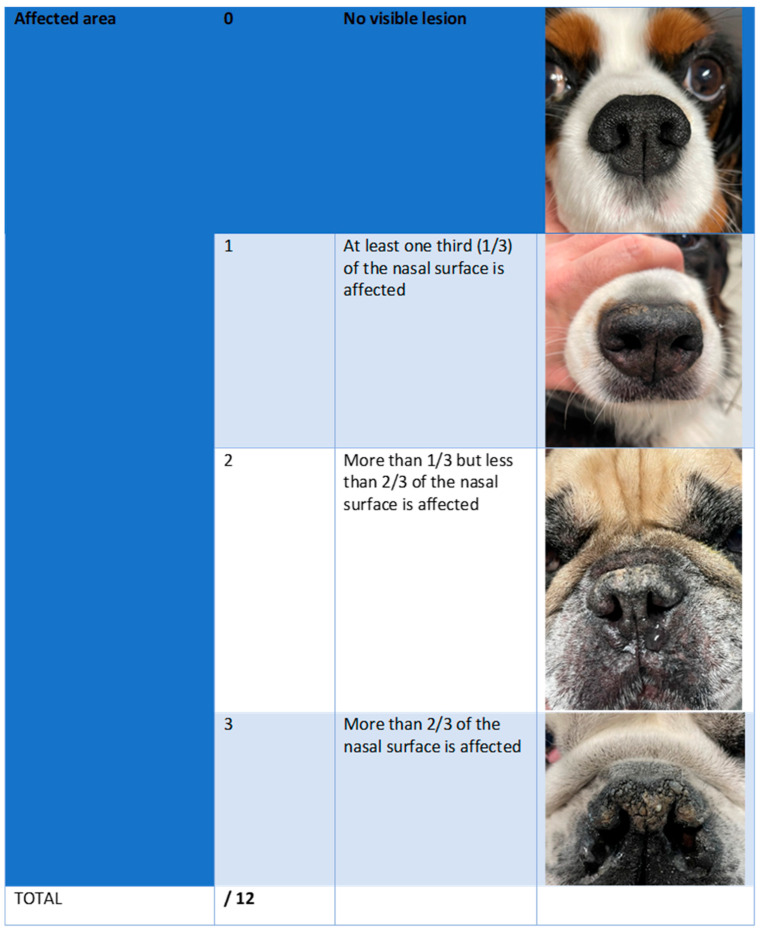
Global dermatological score. This composite score is composed of four criteria, “dry-ness”, “lichenification”, “presence of crusts”, and “affected area”, each rated from 0 to 3.

Dogs of any breed, sex or age showing clinical signs of familial or idiopathic NHK, characterised by thickening of the stratum corneum, were included if they had a minimum global dermatological score (GDS) of 4 (as described in Figure 1) at baseline.

Non-inclusion criteria were a loss of nose print patterns, presence of cracks or ulcerations, concomitant lesion of the muzzle, secondary infections (purulent exudate), nasal hypopigmentation, mucocutaneous involvement, or impaired general health status. A withdrawal period of 6 weeks was required regarding the administration of retinoids, zinc supplements, steroid anti-inflammatory drugs (SAIDs), or essential fatty acids (EFA). In the same way, any keratomodulating shampoos, emollients, lubricating products, or anti-inflammatory/immunomodulating drugs were forbidden 2 and 3 weeks, respectively, before enrolment.

### 2.2. Treatment Protocol

Selected dogs were all treated with the same tested item corresponding to an emollient ointment composed of vegetal oils, fatty alcohols, emollient and restructuring agents, and vegetal extracts (Sensiderm^®^ Balm, MP Labo, Grasse, France).

Owners were instructed to apply the ointment twice daily for 60 days in a thin, uniform layer over the entire nasal planum. While a standard pea-sized amount (approximately 0.5 g) was recommended, the actual quantity applied was adapted to the size of each dog’s nose, leading to potential variations in the amount used. To promote optimal absorption and reduce product loss through licking, owners were advised to gently restrain the dog and keep its mouth closed during and shortly after application. Any new systemic medication with a potential impact on the clinical presentation or any topical product other than the tested item was forbidden during the study.

### 2.3. Efficacy Outcomes

At each follow-up visit (Days 0, 30 ± 2, and 60 ± 2), a general physical examination was performed, during which the overall condition of the dog was rated as good, fair, or poor. For consistency, each dog was evaluated by the same investigator and the same owner throughout the study period. Both the investigator and the owner independently assigned a global dermatological score based on their clinical assessment of the dog’s nasal planum.

A specific grid was especially developed for this study and consisted of four criteria (dryness, lichenification, presence of crusts, and affected area), each rated from 0 to 3 (Figure 1). Each item was illustrated with a representative photograph to guide the observer. The total score ranged from 0 to 12. The four selected criteria were based on the most commonly observed clinical signs associated with idiopathic nasal hyperkeratosis in dogs and were partially adapted from the evaluation parameters described by Catarino et al. [10]. The criterion of suppleness, although relevant, was intentionally excluded as it relies on tactile perception and thus introduces a subjective sensory bias that could not be standardised through visual assessment alone. Both crusting and lichenification were retained as separate items, despite potential clinical overlap, to determine which of the two would prove more intuitive and relevant for both veterinary and non-veterinary observers, given that the concept of lichenification may be less familiar to laypersons. For each criterion and each score level, reference photographs were selected blindly from a pool of several dozen nasal images by three independent veterinarians, ensuring objectivity and consistency in the visual scoring system.

Using a visual analogue scale (from 0 for “very poor” to 10 for “very good”), a pseudo-quantitative evaluation of the clinical evolution was performed on Day 30 and Day 60 by both investigators and owners. A mycological culture, or alternatively any other complementary test that could help guide the diagnosis, was at least recommended if the veterinarian assessed the evolution as 3 or below. Moreover, owner discomfort was evaluated on Day 0, whereas dog discomfort was evaluated each time using a visual analogue scale from 0 (for “I am not/my dog doesn’t seem to be uncomfortable”) to 10 (for “I feel/my dog seems to be very uncomfortable all the time”).

Owners also rated their satisfaction regarding the efficacy of the product on Day 30 and Day 60, on a 0 to 10 point visual analogue scale. At the end of the study, they were asked to rate their global satisfaction on visual analogue scale from 0 (for “I am not satisfied at all”) to 10 (for “I am extremely satisfied”). Additional pieces of information (e.g., presentation of the tube and consistency of the product) were requested from them as well during the follow-up period.

The primary outcome was defined as the evolution of the mean global dermatological score over time. The correlation between investigator and owner evaluations was also analysed. Secondary efficacy outcomes included the mean percentage improvement of the global dermatological score, the proportion of dogs presenting with a healthy nasal appearance (defined as a global dermatological score of 3 or less), the improvement of each individual dermatological criterion, and the pseudo-quantitative evaluation of clinical signs—each assessed by both investigators and owners on Day 30 and Day 60. The definition of a healthy nose was based on the initial pool of nasal photographs, which were used to determine the reference photographs, that exhibited total scores ranging from 0 to 3 and appeared macroscopically normal. However, to be classified as healthy, no individual criterion score could exceed 1.

The evolution of animal discomfort over time and the different owners’ satisfaction scores were also assessed.

### 2.4. Safety Evaluation

Tolerance was assessed by all observers over time. Any severe side effects had to be reported as soon as possible to the investigators.

### 2.5. Statistical Methods

The mean clinical scores were compared between Day 30 and Day 0 and between Day 60 and Day 0 using a generalised linear mixed effects model (with “time” as a fixed effect and “animal” as a random effect). When significance was reached, pairwise comparisons were performed using Fisher’s least significant difference (LSD) post hoc procedure. McNemar’s test was used to compare the percentages of animals with normal and abnormal noses on Day 30 and Day 60 vs. Day 0. A Sign test was used to compare the percentage improvement of the total clinical scores and the different investigator/owner assessments over time. The significance level for all tests was set at 5%.

## 3. Results

### 3.1. Animals

A total of twenty dogs were enrolled in the study. All dogs fulfilled the inclusion criteria, and none were excluded during the study. Of the 20 dogs included in the study, 12 were males and 8 were females, from 3 to 16.2 years of age, with an average age of 8.1 (±3.4) years.

Brachycephalic breeds were overrepresented, with French Bulldogs being the most frequent (*n* = 12; 60%), followed by Boxers (*n* = 2; 10%), English Bulldogs (*n* = 1; 5%), and American Cocker Spaniels (*n* = 1; 5%). The remaining dogs included one German Shepherd, one Belgian Shepherd, and one crossbreed dog.

The mean duration of clinical signs prior to enrolment in the study was 3.6 (±2.4) years and varied from one month to 8 years. The duration of signs prior to D0 was reported as “several years” in one dog and therefore could not be included in this specific analysis.

### 3.2. Outcome

**Figure 2:**
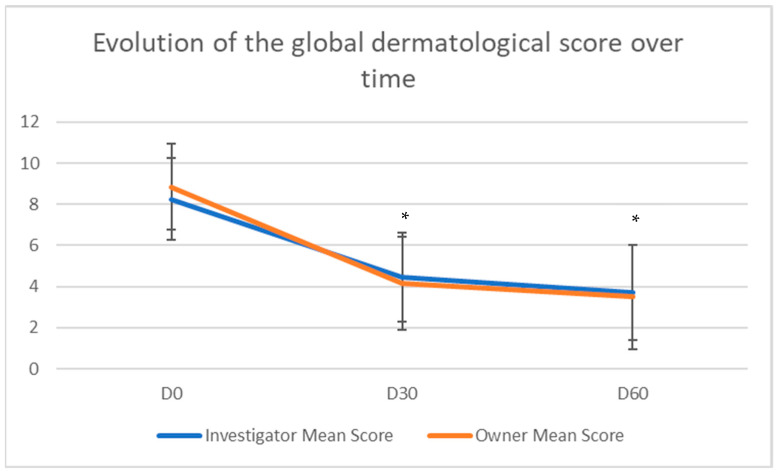
Primary efficacy outcome corresponding to the evolution of the mean global dermatological score, following investigators’ and owners’ assessments. The scores were significantly lower on Day 30 and Day 60 compared to Day 0. * *p* < 0.0001.

**Figure 3:**
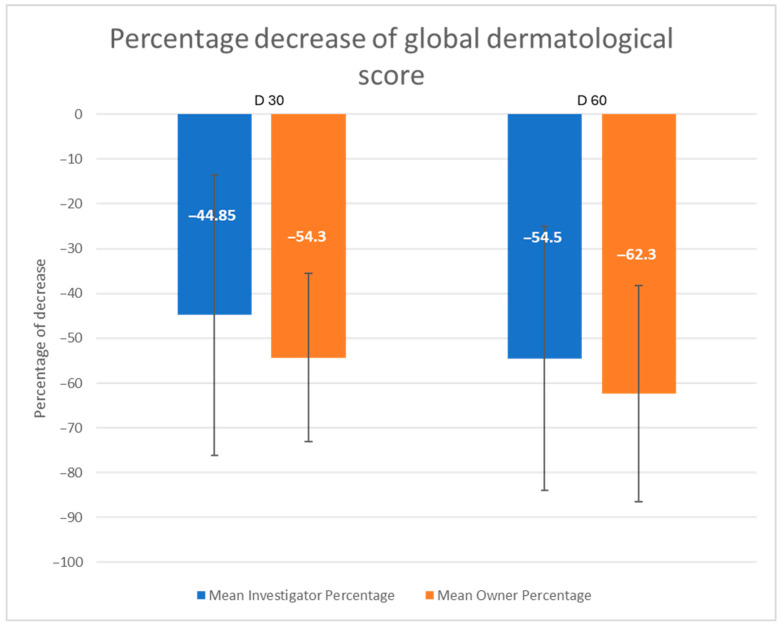
Percentage decrease in the global dermatological score compared to D 0, following investigators’ and owners’ assessments.

All dogs were in a good health condition on Day 0, except one dog suffering from concurrent severe osteoarthritis. The GDS decreased significantly and steadily over time for both investigators and owners (Figure 2). It was significantly lower on Day 30 and Day 60 compared to Day 0 (*p* < 0.0001) and was significantly correlated between observers (*p* < 0.05). After 30 days, the GDS had more than halved, according to owner evaluations. Indeed, the percentage decrease (Figure 3) reached 44.9% and 54.3% on Day 30, and 54.5% and 62.3% on Day 60, for investigators and owners, respectively. Though there was no statistically significant difference between Day 30 and Day 60, the clinical improvement persisted until the end of the trial.

**Figure 4:**
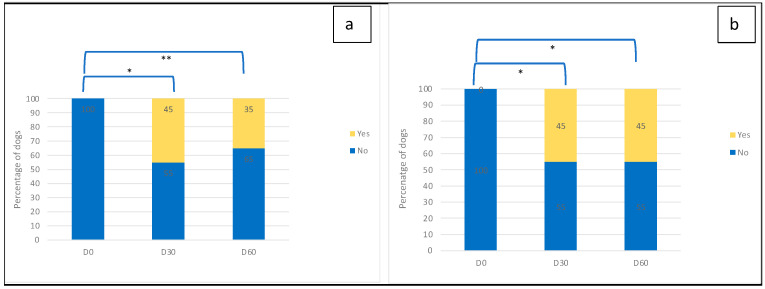
Percentage of dogs with a “healthy” nose (under the limit set for inclusion) (**a**) following investigators’ assessments and (**b**) following owners’ assessments. This percentage was significantly higher for both observers on Day 30 and Day 60 compared to Day 0. MacNemar test, * 0.005 < *p* < 0.010 and ** 0.010 < *p* < 0.025.

**Figure 5:**
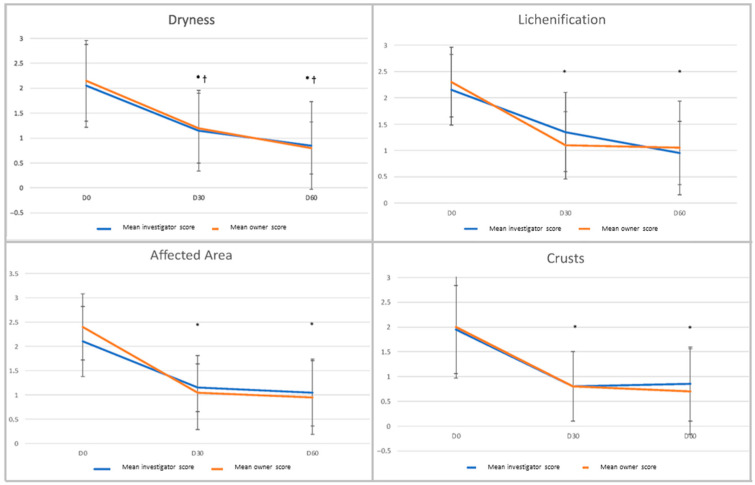
Evolution of the individual dermatological scores (0–3) over time for investigators and owners. Scores on Day 30 and Day 60 were all significantly different compared to D 0. * *p* < 0.0001 and † *p* = 0.0001.

The percentage of dogs with a “heathy” nose was 45% on Day 30 for all observers and 35% and 45% on Day 60 for investigators and owners, respectively (Figure 4). This percentage was significantly higher for all observers on Day 30 and Day 60 compared to Day 0 (*p* < 0.010 on Day 30 for investigators and on both Day 30 and Day 60 for owners, *p* < 0.025 on Day 60 for investigators). “Dryness”, “lichenification” and “affected area” scores decreased significantly and steadily over time compared to Day 0 (Figure 5) for both investigators and owners (*p* < 0.0001 and *p* = 0.0001 for investigators’ assessment of dryness). Despite a mild increase at the end of the trial (Figure 5), the “crust” score was significantly lower on Day 30 and Day 60 compared to Day 0 (*p* < 0.0001) when assessed by the investigators. When evaluated by owners, the decrease in the score was also constant over time and significantly different from Day 0 (*p* < 0.0001). The correlations between observers’ assessments were statistically significant at all time points for “dryness” and “affected area” and only on Day 60 for “crusts” (*p* < 0.05). No correlation was found between investigators and owners for the “lichenification” score. The mean scores for clinical evolution were 8.0 (±1.4) and 7.4 (±1.8) on Day 30 and 7.2 (±1.8) and 7.5 (±2.3) on Day 60 for investigators and owners, respectively. The animal discomfort score was 3.8 (±3.1) on Day 0 and more than halved after one month, with a score of 1.2 (±1.5) on Day 30 and 1.5 (±2.8) on Day 60. The scores on Day 30 and Day 60 were significantly different compared to Day 0 (Sign tests, *p* = 0.0005 and *p* = 0.006, respectively). However, the trend between Day 30 and Day 60 was not statistically significant (Sign test, *p* = 0.2673).

Mean owner satisfaction with the product’s efficacy achieved high scores, with an average rating of 8.3 (±1.5) on Day 30 and 7.9 (±2.2) on Day 60. After one month of use, 40% of owners rated their satisfaction with the product’s efficacy as 9 or 10 out of 10. The overall satisfaction score at the end of the study was 8.1/10. Additionally, owners expressed positive feedback regarding the product’s tube format (8.6/10), product’s texture (8.85/10), and tube’s duration (8.175). On average, they used 32.5 g of the product over the 60-day study period, with individual usage ranging from 3.5 g to 72 g. Only two of twenty owners had to use a second tube over the 60-day study duration.

### 3.3. Safety

No side effects were reported during the study. The product was therefore considered safe.

## 4. Discussion

This open-label, uncontrolled, prospective pilot trial suggests that Sensiderm^®^ Balm (MP Labo, Grasse, France) may offer clinical benefits in the management of idiopathic nasal hyperkeratosis in dogs. While the results are encouraging, they remain preliminary and should be interpreted with caution due to the limited sample size and the absence of a control group or blinding. These findings are hypothesis-generating and warrant confirmation through larger, placebo-controlled, and double-blinded clinical studies.

In the study by Catarino et al. [10], brachycephalic breeds such as bulldogs were the most represented breeds among recruited animals. In our study, brachycephalic dogs represented 75% of the dogs that completed the trial compared to 55% (*n* = 26) of French bulldogs and 15% (*n* = 7) of English bulldogs in the Catarino study [10]. These findings seem to corroborate the idea that brachycephalic breeds are predisposed to NHK [9].

The improvement of clinical signs may result from the original combination of ingredients. Adequate hydration is crucial for skin renewal, as a low water content can impair the breakdown of proteins like desmoglein 1, desmocollin 1, and corneodesmosin [11]. Urea and glycerin, both hygroscopic humectants, enhance water retention within the stratum corneum, while shea butter, an emollient, limits transepidermal water loss [11,12,13]. In addition to their moisturizing properties, glycerin and panthenol (dexpanthenol) have demonstrated significant regenerative effects and wound-healing capacities in various dermatological conditions [14,15,16,17]. Urea and panthenol may also contribute to epidermal homeostasis through the upregulation of key differentiation genes such as filaggrin and loricrin [12,15]. *Centella asiatica*, a medicinal plant rich in pentacyclic triterpenoids (notably asiaticoside and madecassoside), has shown therapeutic efficacy in vitro, in animal models, and in human patients with skin disorders, including acne, burns, atopic dermatitis, and wounds [18,19]. Furthermore, the mechanical action of gently massaging the balm during application may have helped loosen and remove excess keratin while enhancing hydration independently of the product’s biochemical properties.

In our study, the daily use of the balm for 60 days led to decreases in the GDS of 44.9% and 54.3% on Day 30 and 54.5% and 62.3% on Day 60 for investigators and owners, respectively. The score used by Catarino et al. [10] was slightly different and included four criteria, “dryness”, “suppleness”, “extension” and “lichenification”, with an aggregate score ranging from zero to eleven. In this study, the evaluation, performed by the same investigator, reported a 36.8% reduction in the lesion score after 60 days of product use. One report described a possible improvement in hyperkeratotic lesions (of footpads) within 5 days after daily foot soaks with 50% propylene glycol [20]. Nevertheless, it was not specified when the 50% reduction of hyperkeratosis was obtained. In our study, an improvement in clinical signs was observed after the first 30 days of application. Based on previous findings of Catarino et al. [10], we were expecting a slower improvement of the nasal lesions. During the second month, the progression of improvement was more moderate, and a stabilisation of clinical signs was observed. This plateau effect may reflect the achievement of maximal improvement for each individual case, beyond which further changes became more subtle and therefore more difficult to detect through clinical evaluation alone. Additional clinical studies are needed to determine the precise timing of this stabilisation and to define the optimal frequency of application for long-term maintenance. However, this factor is likely to be dog-dependent, as marked inter-individual variations were noted in both the degree and speed of the clinical response. Consequently, therapeutic decisions should be tailored to each case based on the clinician’s assessment.

The development of the new clinical lesion scoring system was guided by the dual objective of accurately capturing relevant clinical signs while ensuring ease of use for both trained investigators and laypersons (i.e., dog owners). Among the four criteria, “dryness” and “affected area” were consistently rated with strong agreement between investigators and owners at all time points, suggesting that these features are easily recognizable and reproducible. The “crusts” criterion showed a correlation only at the final evaluation, possibly reflecting both improved recognition by owners over time and the fact that, as most dogs improved progressively throughout the study, lesions became less severe and therefore easier to assess reliably by the end of the trial. In contrast, no significant correlation was observed for the “lichenification” score at any time point, despite the use of standardized reference images. This finding highlights the difficulty laypersons may have in identifying or distinguishing this dermatological feature, particularly from crusts. Given its limited inter-observer reliability, especially outside a clinical setting, we recommend that the lichenification criterion be excluded from future scoring grids intended for use by pet owners. This lack of correlation may introduce a degree of variability in the interpretation of treatment efficacy, particularly when relying on owner-assessed scores, and should therefore be considered when interpreting the robustness of the clinical outcomes.

Adherence to the treatment protocol was excellent, despite the requirement for twice-daily applications. Owners reported that the product was easy to use, largely due to its convenient tube packaging and appropriate consistency, which facilitated routine application. The quantity needed per dog was minimal, with an average of only 32.5 g used over the 60-day period, yet a notable clinical improvement and reduced animal discomfort were already evident by Day 30 and continued through Day 60, underscoring the product’s effectiveness in a practical, daily care setting. Nonetheless, variations in the amount of balm applied were observed, as reflected in product consumption data, suggesting heterogeneity in treatment exposure among dogs. Such variability may have influenced the consistency of clinical responses. Additionally, although owners were instructed to gently restrain their dog and keep the mouth closed during application to limit immediate licking and enhance local absorption, this measure did not entirely prevent post-application licking. As a result, partial ingestion or mechanical removal of the product could not be ruled out and may have reduced its efficacy in some individuals. During the study, no safety concerns were reported by any of the observers. This favourable outcome is consistent with the existing literature on the safety profiles of the individual ingredients used in the formulation [13,21,22,23,24,25,26]. In the study by Catarino et al. [10], minor adverse events such as alopecia and erythema around the nasal area were observed in 11.5% of dogs, possibly indicative of contact dermatitis related to the presence of limonene in cajeput essential oil. Additionally, nearly one third of owners (seven owners; 29%) expressed dissatisfaction with the product in that study, citing its strong odour, the dogs’ aversion to application, or the greasy and unstable consistency of the balm at higher temperatures. While direct comparisons should be interpreted with caution given the differences in study design and sample characteristics, the formulation of Sensiderm^®^ Balm—with its use of plant-based extracts, omission of perfumes, and stable texture—appears to be well tolerated. These formulation choices were intended to minimize the irritation of already compromised skin [27] and to preserve the dog’s olfactory comfort. Notably, the absence of fragrance did not negatively affect owner satisfaction, with a high average rating of 7.9/10, reinforcing the relevance of avoiding unnecessary additives in topical products intended for dermatologically sensitive animals.

## 5. Conclusions

This pilot study suggests that the tested emollient balm (Sensiderm^®^ Balm, MP Labo, Grasse, France) may be a valuable option for the supportive management of idiopathic nasal hyperkeratosis (NHK) in dogs. Noticeable improvements in clinical signs were observed as early as one month, with effects maintained over the 60-day study period. In addition to these clinical improvements, a reduction in discomfort and enhanced nasal appearance were reported by owners, contributing to a perceived improvement in patient well-being and quality of life—outcomes that were reflected in high owner satisfaction. The newly developed dermatological scoring grid showed promise in facilitating evaluation, although further refinements are needed to enhance its applicability, particularly for use by non-veterinarians. While the balm was well tolerated, these preliminary findings must be interpreted with caution due to the limited sample size, open-label design, and lack of a control group. Larger, controlled clinical trials are required to confirm the observed trends, evaluate long-term efficacy, and define evidence-based recommendations for routine use.

## Data Availability

The data are available upon request to carole.gard@destaing.com.

## Abbreviations

GDS	Global Dermatological Score
NHK	Nasal Hyperkeratosis
VAS	Visual Analogue Scale
